# Wall-Corner Classification Using Sonar: A New Approach Based on Geometric Features

**DOI:** 10.3390/s101210683

**Published:** 2010-11-30

**Authors:** Milagros Martínez, Ginés Benet

**Affiliations:** Instituto de Automática e Informática Industrial *(ai2)* 1, Universidad Politécnica de Valencia, C/. de Vera, s/n, 46022, Valencia, Spain; E-Mail: mimar@disca.upv.es

**Keywords:** target classification, target localization, map building, ultrasonic sensors, classification algorithms

## Abstract

Ultrasonic signals coming from rotary sonar sensors in a robot gives us several features about the environment. This enables us to locate and classify the objects in the scenario of the robot. Each object and reflector produces a series of peaks in the amplitude of the signal. The radial and angular position of the sonar sensor gives information about location and their amplitudes offer information about the nature of the surface. Early works showed that the amplitude can be modeled and used to classify objects with very good results at short distances—80% average success in classifying both walls and corners at distances less than 1.5 m. In this paper, a new set of geometric features derived from the amplitude analysis of the echo is presented. These features constitute a set of characteristics that can be used to improve the results of classification at distances from 1.5 m to 4 m. Also, a comparative study on classification algorithms widely used in pattern recognition techniques has been carried out for sensor distances ranging between 0.5 to 4 m, and with incidence angles ranging between 20° to 70°. Experimental results show an enhancement on the success in classification rates when these geometric features are considered.

## 1. Introduction

Sonar sensing is one of the most useful and cost-effective methods of sensing. Sonar sensors are light, robust, and inexpensive devices. These reasons have led to their widespread use in applications such as navigation of autonomous robots, map building, and obstacle avoidance. Although sensors provide accurate information about locating and tracking targets, they may not provide by themselves complete information about the nature of detected objects, which is the reason for combining data from multiple sensors using data fusion techniques. The primary aim of data fusion is to combine data from multiple sensors to perform inferences that may not be possible with a single sensor. Fusion techniques allow performing combination of different measurements from same sensor, but taken from different points of view.

The widely used Polaroid sensor does not provide the echo amplitude directly from the available commercial boards. Thus, most sonar systems use time of flight (TOF) and the bearing angle as the only information source to classify the targets [1]. In [2] a single Polaroid sensor is used to differentiate between edges, planes and right corners. The edges are differentiated from plane/corner using only one measurement. Planes are differentiated from corners by taking two measurements from two separate locations.

In [3] two piezo-ceramic US sensors, located at the same site, were used to distinguish between walls and corners in a single step, and a new multi-echo ultra-fast firing method increased the sonar acquisition rate, and provided crossed measurements without interference. A tri-aural sensor arrangement which consists of one transmitter and three receivers is proposed in [4] to differentiate edges/right corners/planes using TOF. A similar sensing configuration is proposed in [5] to estimate the radius of curvature in cylinders. All these techniques have in common the need for obtaining high precision in the measurements, measuring from two different locations (or have an array of at least two sensors) in order to obtain a classification of the obstacle. Information from ultrasonic sensors has poor angular resolution, which is why a single sensor is not enough to distinguish between wall or corner using the above indicated techniques.

Other approaches make use of dedicated hardware using DSP and multiple transmitter-receiver ultrasonic pairs to enhance the classification results and the time response of the sonar system. In [6], a sonar system implemented with a DSP, 2 transmitters and 2 receivers has been reported. The sonar system delivers accurate range and bearing measurements with interference rejection. The classification approach operates in two sensor cycles, by alternately firing one transmitter and then the other, allowing a moving sensor to perform classification into walls, corners and edges. The classification is based on virtual images and mirrors. Same authors, in [7], compensated the effects of linear velocity of the robot on the TOF and reception angle for the three obstacle types, allowing a moving sensor to perform robust classification at speeds above 0.6 m/s in scenarios with statics objects. In [8] and [9] a robot with an advanced sonar ring using 24 transmitters and 48 receivers is described, which accurately measures range and bearing. However object classification cannot be made within only one measurement cycle and requires consecutive readings to confirm the classification using Kalman filters.

In [10] an array of transducers with 2 emitters and 4 receivers is described. This enables the reduction of the scanning time by means of simultaneous transmission in the emitters. Again, only range and bearing is used. The system provides 8 values of TOF after a single measurement cycle, and can classify reflectors into 3 types: walls, corners or edges. The implementation is based on DSP-FPGA architecture, and it is capable of computing all the algorithms in real time.

The use of successive readings to refine the classification results is also common and there are many papers that use data fusion algorithms to construct maps in real time. The paper [11] describes a technique for the fusion of data obtained from standard Polaroid sonar sensors to create stochastic maps. The main idea is to interpret TOF-only data from multiple uncertain points of view, and using the Hough transform to identify points and line segments.

The references [12] and [13] describe a robot equipped with eight pairs of piezo-ceramic ultrasonic sensors (ring of sensors). These authors focus their work on building a grid map whose primitive features are lines, points and circles, extracted by means of the relationship between two or more individual sonar measurements taken from different points of view. Information used are TOF and bearing only. The features are processed by trimming, division or removal, depending of the dynamic circumstances.

In general, the classification process achieves better ratings when it uses the TOF in addition to the signal amplitude. Unfortunately, amplitude is very sensitive to environmental conditions, as relative humidity, temperature, air pressure, *etc*. Some authors use data fusion techniques to reduce the uncertainty in the measurements. In [14] Dempster-Shafer evidential reasoning and majority voting were used to fuse the data obtained from an object from two different points. Also, in [15] and [16] the same authors extended their previous works by using pattern recognition techniques in the classification.

In [17] a feature-based probabilistic map is built using TOF and amplitude of a sonar. The amplitude reduces the ambiguity due to beam width, and reduces the number of measurements. Target types are walls, corners and edges. Extended Kalman filters and Bayesian conditional probabilities are also used to enhance the final maps produced.

A broadband, frequency-modulated sonar sensor for generating maps is described in [18]. This paper uses both amplitude and TOF to extract point-targets on both smooth and rough surfaces. The availability of amplitude allows them not only to estimate target type but also recognize different types of surfaces. The mapping process fuses target observations and the result is a map of geometric primitives (lines or points). Extended Kalman filter is used to refine the results and remove the dynamic objects from scene.

In [19] an array of Polaroid sensors is used in order to distinguish between planes and corners by using both amplitude and TOF information. In [20] the same authors extended the algorithm to distinguish more obstacles, and in [21] and [22] a neural network is added to enhance the classification results. In [23] a single Polaroid sensor is used to classify between four types of obstacles using TOF, amplitude and frequency of the signal.

As previously indicated, many authors classify the objects found in a scene into 3 main object classes: walls, corners and edges. However, taking into account that we use piezo-ceramic transducers, (cheaper than Polaroid, but less sensitive), only the first two classes (walls and corners) are found in our experiments. This is due to the fact that edges have a very reduced reflector area, and consequently, their echoes are of very reduced amplitude. Thus, their resulting peaks can be confused with noise, and they are not detected. In our opinion, this reduction of the object classes to walls and corners is sufficient to model satisfactorily the real world found in our experiments.

This paper presents and discusses a new set of features derived as a consequence of the corner’s geometry. These new features can be used by different classification algorithms to enhance the wall/corner classification results. Thus, a comparative study between the most usual classification algorithms has been carried out, and their results are presented in this paper using these new geometric features as well as other features obtained directly from the echo.

Also, it must be emphasized that the classification algorithms discussed in this paper use only data obtained from a single sonar scan taken from the same position. Thus, these classifications do not depend on previous classifications for the same objects found in previous sonar scans. Consequently, the final map can be further enhanced using any of the data fusion techniques used in some of the above described papers, but this aspect is out of the scope of the present paper.

The paper is organized as follows: Section 2 summarizes our previous work. Section 3 presents the new geometric features derived from the ultrasonic echo and proposes a method for obtaining them. Section 4 presents a classification algorithm that uses only the amplitude model, taking it as a basis for comparison. Section 5 describes three algorithms that are based on pattern recognition techniques, and combine the information obtained using the amplitude model and geometric information contained in the echoes taken from the corners. Section 6 presents the results of testing the surfaces of various materials at different distances (1.5 m to 4 m) from the robot, as well as the results obtained in the wall/corner classification by applying all the algorithms described in this paper. Finally, conclusions from these results are discussed in the Section 7.

## 2. Previous Work and Scope

**Figure 1.**
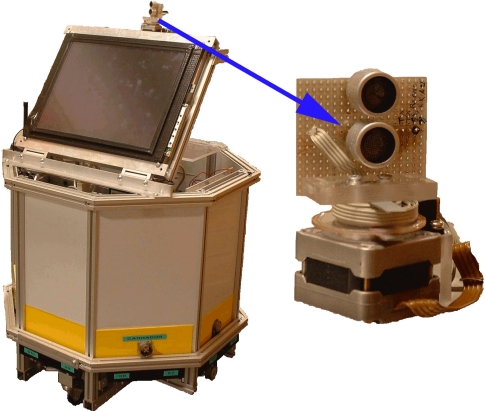
YAIR robot prototype. The sonar sensor can be seen in more detail at the right part of the figure. The transmitter and the receiver are piezo-ceramic transducers vertically aligned and driven by a stepper motor.

In our experiments we used the robot YAIR [24]. Figure 1 shows this robot together with the rotary ultrasonic sensor on its top. The sensor has two transducers: one transmitter and one receiver, enabling the two transducers to have the same rotating axis. This rotating array is driven by a stepper motor with 1.8 degrees per step, and is able to get up to 200 angular samples in each scan. In each angular position, the transmitter sends a train of 16 ultrasonic pulses and normally up to 256 samples of this signal are recorded before the stepper motor advances to the next position, repeating the process. Thus, in each angular position a vector of 256 samples is stored and processed, and the complete scan will produce an array of 200 angular echoes. It should be taken into account that for each angular position the amplitude of the ultrasonic echo is available and subsequently, information about the situation of obstacles can be calculated later by using the TOF method.

**Figure 2.**
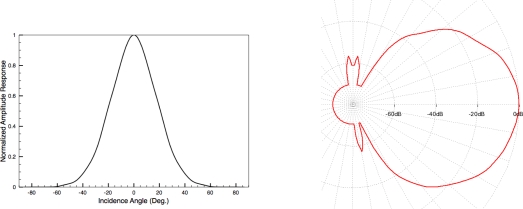
Peak amplitude versus incidence angle using piezo-ceramic ultrasonic transducers. Left: linear plot; Right: polar plot.

Also, it must be noted that ultrasonic sensors usually have relatively wide sensitivity cones. This means that obstacles separated up to an angle *θ*_0_ from frontal orientation of the sensor can produce appreciable peak amplitudes in the received echo. In the case of the piezo-ceramic transducers used in our work, this angle *θ*_0_ is about 55°. In Figure 2 the angular amplitude response of our ultrasonic transducer is shown, presented both in linear (left) and polar (right) plots. These two plots show us that a single object will produce an amplitude peak corresponding to the distance from this object to the sensor during 110 degrees of rotation of the sensor. Of course, the maximum value of these registered peaks will correspond with the frontal orientation between transducers and object.

The amplitude model presented in [25] and [26] exploited the differences between the amplitude taken from a wall (flat surface) and the amplitude taken from a right-angled corner (formed by two orthogonal walls). By applying the model of amplitude it is possible to differentiate between the echo taken from a wall, which has only one reflection of the signal, and the echo taken from a right-angled corner, with two reflections of the signal, corresponding to each generator-wall. This classification system offers good results, but still has some inconveniences, as will be detailed later in Section 4. To circumvent these problems, in this paper a new set of features based on the geometric properties of the corners is presented. These geometric features will be used as described next in Section 3. Using these features as an input, some well-known pattern recognition techniques can be used to refine the overall classification process.

Finally, a comparative study of different methods in wall/corner classification is presented, taking the amplitude model algorithm as a reference and using the proposed geometrical features. The elected algorithms to be compared in our study are:

1. Amplitude model-based classification algorithm.
2. K nearest neighbor (KNN).
3. Prototyping of Denoeux combined by Dempster-Shafer evidential reasoning (Denoeux-DS).
4. Quadratic discriminant analysis (QDA).

## 3. Extracting Knowledge from the Geometric Characteristics of the Environment

**Figure 3.**
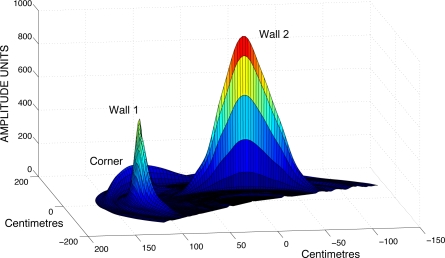
3D representation of the intensity amplitude obtained from a corner. The figure shows that the corner-generator walls 1 and 2 return higher peak intensities than corner C.

**Figure 4.**
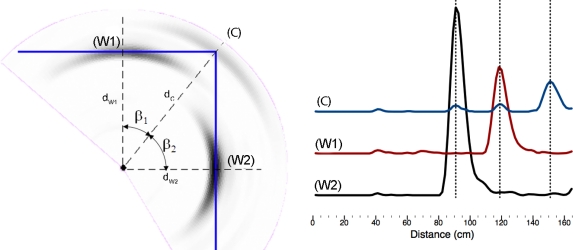
Echoes returned from the corner of Figure 3. Left: 2D representation of the circular scan. Right: received echoes corresponding to the three peak amplitudes (*Wall*_1_, *Wall*_2_ and *Corner*).

After an entire circular scan has been performed with the ultrasonic sensors, we get 200 echoes, each one corresponding with an angular position of the rotary sensor. These echoes represent the amplitudes of the received signal as a function of the elapsed time from the beginning of the echo. Taking into account that the ultrasound speed is constant, it is easy to plot the received amplitude as a function of the distance, instead of time. Figures 3 and 4 show the received amplitudes as a function of distance corresponding to a right-angled corner. Each amplitude peak corresponds with an object of the scenario (wall or corner).

The amplitudes taken from each of the generator walls are higher than the amplitude returned from the corner. A representation of the maximum intensity points of each echo shows an arc for each obstacle, as shown in Figure 4. The maximum value of each arc corresponds to the obstacle: wall or right-angled corner, and it is placed at incidence angle normal to the surface. The echoes taken from a wall (point *W*_1_ or *W*_2_) only have a maximum peak, corresponding with the distance *d*_*w*1_ or *d*_*w*2_. However, the echo taken from the corner (*C*) is different. In Figure 4 the echo of the corner has two “ghost peaks” of lower intensity than the main maximum, one corresponding to the distance *d*_*w*1_, and the other to the distance *d*_*w*2_. The main maximum, which corresponds with the corner distance *d_c_* is the higher one. This difference of shape in the amplitude plots can be used to differentiate between corners and walls.

In summary, when the surfaces that form a corner are sufficiently large, they produce in their echo in the corner two “ghost peaks” that appear before the corner’s peak and correspond with distances *d*_*w*1_ and *d*_*w*2_. Note that angles *β*_1_ and *β*_2_ are complementary (*β*_1_ + *β*_2_ = 90°) and can be expressed as:

(1) $$\beta_{1}=\text{arccos}(\frac{dw1}{dc});\;\;\;\text{and}\;\beta_{2}=\text{arccos}(\frac{dw2}{dc})$$

These ghost peaks are due to the ultrasonic sensor wide angular sensitivity beam of 2*θ*_0_ degrees. For this reason, any obstacle found between this angle will produce a peak of amplitude. In our case, most piezo-ultrasonic sensors have *θ*_0_ = 55°. As previously indicated, this means that any obstacle, such as a wall, provides an echo during an angular interval of 110°, returning the maximum amplitude when the incidence angle of the sensor is normal to the surface of the obstacle. The wide angular response of the sensor is the responsible of this phenomenon, as can be seen in Figure 2.

### Special cases

There are several instances in which the echoes of corners have no *“ghost peaks”*. In following subsections we will describe three situations that produce this effect.

### Far corner

**Figure 5.**
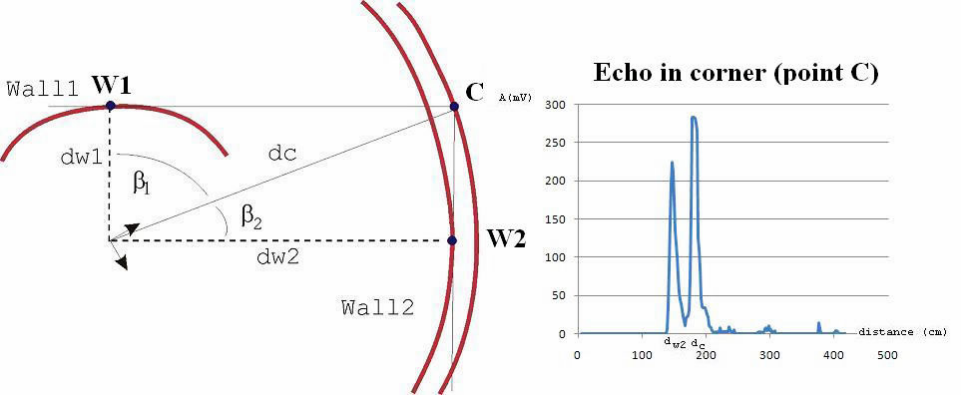
Single “ghost peak”. Case of far corner. Angle *β*_1_ is bigger than 55°. The echo in the corner has only the interference of *wall*_2_ (*β*_2_ < 55°).

If any of the angles *β*_1_ or *β*_2_ exceeds value *β*_0_ = 55° then there is no appreciable wall echo from its corner, as shown in Figure 5. The echo in the corner has only a “ghost peak”.

### Equidistant Corner

**Figure 6.**
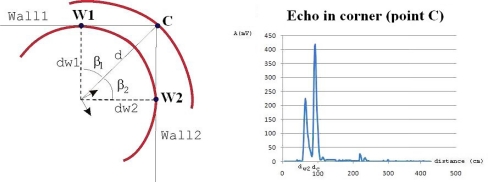
Transducer placed in the bisector of the corner. Echoes of the walls overlap at the same distance, appearing only one “ghost peak” before the corner’s peak.

This situation is produced when transducer pair is equidistant from both generator-walls. In this case the angles *β*_1_ = *β*_2_ = 45° and the walls are at the same distance (see Figure 6). Thus, their echoes overlap and there appears only one “ghost peak” before the main corner peak.

### Corner with a Pillar

**Figure 7.**
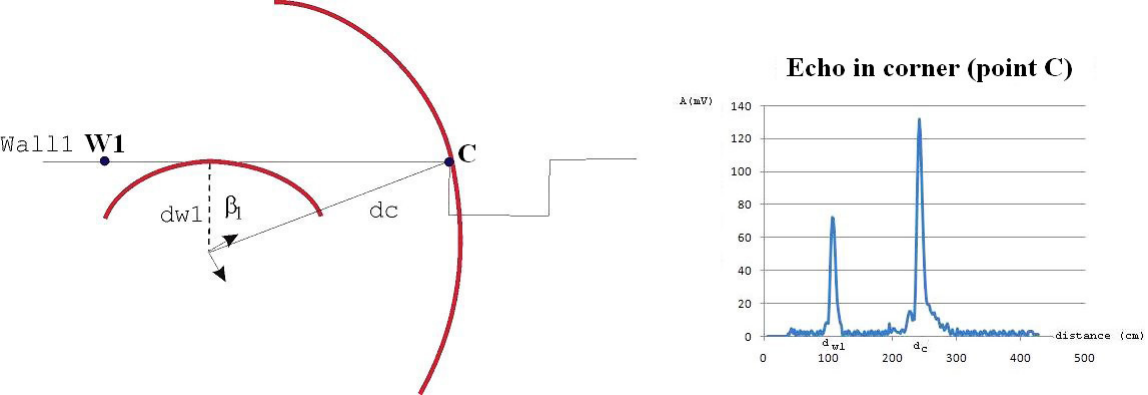
Corner produced by a protruded pillar.

This case occurs typically when a squared pillar protrudes slightly from a wall. This corner is produced by a big wall (main wall) and a little wall (one of the sides of the pillar). In this situation usually only one “ghost peak” can be observed in the corner’s echo, as is evident from Figure 7. In general, when one of the two walls are not within the field of view of the transducers, the echo of their corner will miss the corresponding “ghost peak”.

## 4. Classification Procedure Based on Amplitude

The echoes taken from a wall are different from the echoes taken from a corner. The first difference is the amplitude of the main local maximum, which corresponds to the main obstacle (wall or corner). As previously described in [25] and [26] this information is useful to obtain a first classification as wall or corner, by using the amplitude model:

(2) $$A_{i}=A(d,\;\beta_{i},\;N)=A_{0}C_{r}^{N}\frac{e^{-2\alpha d}}{2d}e^{\frac{-4\beta_{i}^{2}}{\theta_{0}^{2}}}$$

where:

*N* is the number of reflections of the signal,
*d* is the distance to the obstacle,
*β_i_* is the incidence angle to the obstacle,
*A*_0_, *α*, *θ*_0_, *C_r_* are model parameters (constants).

The parameter *N* will be calculated from the previous equation, assuming incidence angle *β_i_* = 0 as follows:

(3) $$N=\frac{\text{ln}\left(\frac{2A_{i}d}{A_{0}}\right)+2\alpha d}{\text{ln}(C_{r})}$$

By comparing a large amount of walls and corners, it is reported in [26] that the parameter *N* follows a normal distribution, centered on 1 for the walls and on 2 for the corners, with standard deviations that depend on the composition of the environment’s surfaces. Thus, an object is classified as a wall if the value of this parameter *N* obtained from Equation (3) gives a value under a threshold *N*_0_ (in [26] a value of *N*_0_ = 1.5 is used, and normal distributions with the same standard deviation *σ* = 0.33 were found satisfactory to simulate the experimental results). If the calculated value of *N* is over this threshold *N*_0_, the object will be classified as a corner. This is a very straightforward procedure of classification that offers very good results at short distances and with scenarios made with the same material: 88% success rate on average in walls, and 85% in corners (for distances under 1.5 m). Also, the membership probability for each class can be derived from the gaussian probability distribution properties, yielding the following exponential sigmoid:

(4) $$\begin{array}{l} P(\text{wall})=\frac{1}{1+e^{\frac{N-N_{0}}{\sigma^{2}}}}\\ P(\text{corner})=1-P(\text{wall}) \end{array}$$

However, if the scenario is a corner made with two walls of different materials, the above classification method will fail, because it is based on the assumption of the same surface material for all the scenario. This explains why the success rate of classification of the corners in these cases is reduced down to 70%. Moreover, at long distances, classification is not as good as for the corners. From 1.5 m to 4 m the success rate is over 55% in corners, however in walls it remains the same value: 88%. Long distances (range 1.5 m to 4 m) and different kinds of materials are the worst combination, and the success rate in these cases for walls is about 67%, and over 40% in corners.

## 5. Classification Algorithms Based on Pattern Recognition Techniques

Pattern recognition aims to classify data based either on *“a priori”* knowledge or on statistical information extracted from the patterns. The patterns are usually groups of measurements or observations that define points in an appropriate multidimensional space. In this paper, the available set of features obtained from the echoes are: *v* = {*d*, *a*, *N*, *d*_*w*1_, *a*_1_, *β*_1_, *A*_1_, *d*_*w*2_, *a*_2_, *β*_2_, *A*_2_}

Some of these features are obtained directly from the echo:

- *d* distance to the obstacle, (measured from the ToF),
- *a* amplitude of the main maximum located at the distance *d*, (highest amplitude of the echo),
- *d*_*w*1_ and *d*_*w*2_, measured distances of the ghost peaks, (*d*_*w*1_ *< d*) and (*d*_*w*2_ *< d*),
- *a*_1_ and *a*_2_ amplitudes of the ghost peaks at distances *d*_*w*1_ and *d*_*w*2_ respectively.

And the rest of features are calculated as follows:

- *N* number of reflections: 1 walls, 2 corners. It is calculated using the amplitude model (Equation (3)) and the parameters *d* and *a*,
- *β*_1_ and *β*_2_ are incidence angles of the generator walls *W*1 and *W*2 respectively. They are calculated using *d*_*w*1_, *d*_*w*2_ and *d*, (Equation (1)),
- *A*_1_ and *A*_2_ are the theoretical amplitudes of the relative maximum located at distance *d*_*w*1_ and *d*_*w*2_ respectively. These relative maximums probably are caused by generator wall *W*_1_ or *W*_2_. Ideally *A*_1_ (calculated) = *a*_1_ (measured), and the same applies for *A*_2_ (calculated) = *a*_2_ (measured).

Three algorithms, widely used in pattern recognition, have been selected in our work: the “*K-nearest neighbors*” (KNN), the “*Denoeux-Dempster-Shafer*” (Denoeux-DS) and “*quadratic discriminant analysis*” (QDA). Each of them use sub-sets of the above listed features. Main characteristics of these algorithms are described in the following sub-sections.

### 5.1. Application of K-Nearest Neighbors (KNN Algorithm)

The KNN algorithm uses a set of training patterns in order to estimate the probability that an observation belongs to a class. The cost of this algorithm is quite high because it has to calculate the Euclidean distance to each of the neighbors, and sort them to find the nearest K. To implement the algorithm the following standard requirements are required:

1. Number of classes, will be Ω = {wall, corner}.
2. Set of training patterns representative of each class. A set of 300 patterns of walls and 300 patterns of corner is used: *C* = *c*^(1)^, *c*^(2)^, …, *c*^(300)^ and *W* = *w*^(1)^,*w*^(2)^, …,*w*^(300)^.
3. The vector of normalized features is *v* = {*v*_1_, *v*_2_, *v*_3_, *v*_4_}. (Normalized to one).
  (5) $$v_{1}=\frac{N}{2};\;\;v_{2}=\left|\frac{A_{1}-a_{1}}{A_{1}}\right|;\;\;v_{3}=\left|\frac{A_{2}-a_{2}}{A_{2}}\right|;\;\;v_{4}=\frac{90-\beta_{1}-\beta_{2}}{90}$$

The obstacle will belong to the class which belongs to the majority of *K* nearest neighbors. Moreover, the choice of the parameter *K* is another important factor in the ranking, according to the results obtained in our tests, the value of *K* = 10 provides the best results.

### 5.2. Algorithm Based on Prototypes of Denoeux and Unification Criteria for Dempster-Shafer (Denoeux-DS Algorithm)

The “*Denoeux method*” [27,28] establishes a set of prototypes for each class. The algorithmically hard phase is the first, when prototyping is taking place. A set of patterns for each prototype is needed. The set of features is defined, and constants are calculated. The more patterns it calculates the better the prototype fits. Once the prototypes are defined takes place the second phase: the classification. Classification has two steps of calculation:

- the value which means distance or proximity from an observation to each prototype, and then probability of belonging to each class, and
- fusion of probabilities (only a probability for each class). The Dempster-Shafer’s rule is used in order to fuse the knowledge.

#### Establishment of prototypes

The method proposed by Denoeux in[27] is inspired by the allocation of evidence based on Dempster-Shafer method theory [29] according to the proximity of the data to certain prototypes of each class. In some ways it resembles the KNN algorithm, but the algorithmic load is significantly smaller because it is only used in the prototyping phase. Training patterns are only used to define each prototype and their distance function *df_i_*. Later the distance function is used to assign a probability of membership to the class that represents the prototype, Φ*_i_*. As Denoeux method indicates, the parameter *k_i_* is the inverse of the average of *df_i_* (of patterns).

(6) $$\Phi_{i}(df_{i})=e^{-k_{i}df_{i}^{2}}$$

When an obstacle is being classified, initially the distances *df_i_* to all the prototypes are obtained, then the probabilities to each class are calculated, using *P_i_* = Φ*_i_*, and subsequently several results for each class (*P*_1_, *P*_2_…*P_H_*) are obtained, (*H* prototypes, *H* probabilities). Afterwards, these *H* results must be combined using the Dempster-Shafer’s rule, and finally a unique probability of belonging to a particular class appears. In our case, only two classes are to be considered: wall and corner; and we have three prototypes: wall prototype, right-angled corner prototype and amplitude model prototype. Thus, this method is applied as follows:

#### Wall prototype

The ideal wall prototype is characterized having no ghost peaks (*a*_1_ = *a*_2_ = 0). It is defined as a distance function to the prototype of wall *df_w_* as shown in the following Equation (7). A set of 300 corners training patterns are used and the parameter *L* is 10.

(7) $$\begin{array}{l} {df}_{w}=\sqrt{{\left(a_{1}/L\right)}^{2}+{\left(a_{2}/L\right)}^{2}}\\ \Phi_{\text{wall}}({df}_{w})=e^{-{df}_{w}^{2}} \end{array}$$

Basic knowledge (probability of being wall or corner) is calculated as follows:

(8) $$\begin{array}{l} Pw(\text{wall})=\Phi_{\text{wall}}({df}_{w})\\ Pw(\text{corner})=1-Pw(\text{wall}) \end{array}$$

#### Right-angled corner prototype

The ideal corner prototype is developed based on two ghost peaks. If there is only one peak then amplitude *a*_2_ = 0.

(9) $$\begin{array}{l} {df}_{c}=\sqrt{{\left(\left(a_{1}-A_{1}\right)/A_{1}\right)}^{2}+{\left(\left(a_{2}-A_{2}\right)/A_{2}\right)}^{2}}\\ \Phi_{\text{corner}}({df}_{c})=e^{-{df}_{c}^{2}} \end{array}$$

And the probabilities are:

(10) $$\begin{array}{l} P_{c}(\text{corner})=\Phi_{\text{corner}}({df}_{c})\\ P_{c}(\text{wall})=1-P_{c}(\text{corner}) \end{array}$$

#### Amplitude model prototype

The Denoeux algorithm proposes the use of a set of prototypes. Each prototype is used to know the probability that an observation has to belong to each class. Finally, all probabilities for each class will be merged in order to have only one for each class. Amplitude model is another kind of prototype, where *P_A_*(wall) and *P_A_*(corner) are obtained. Our work uses these probabilities as additional criteria in the final fusion.

#### Dempster-Shafer fusion criteria

We have an initial classification with each of the prototypes: *P_c_*(wall), *P_c_*(corner), *P_w_*(wall), *P_w_*(corner), *P_A_*(wall), *P_A_*(corner), and finally we must have only one *P*(wall) and *P*(corner) = 1 − *P*(wall). The final probability is obtained by applying the combination of the Dempster-Shafer rule. Bearing in mind that the operator ⊕ meets associative and commutative properties, the following equation can be obtained:

(11) $$P=P_{c}⊕P_{w}⊕P_{A}=(P_{c}⊕P_{w})⊕P_{A}=P_{c⊕w}⊕P_{A}$$

The combination *P_c_* ⊕ *P_w_* = *P_c⊕w_* is made as follows:

(12) $$\begin{array}{l} P_{c⊕w}(\text{wall})=\frac{P_{c}(\text{wall})P_{w}(\text{wall})}{1-\text{conflict}}\\ P_{c⊕w}(\text{corner})=\frac{P_{c}(\text{corner})P_{w}(\text{corner})}{1-\text{conflict}} \end{array}$$

where *conflict* is defined as:

$$\text{conflict}\;=P_{c}(\text{wall})P_{w}(\text{corner})+P_{c}(\text{corner})P_{w}(\text{wall})$$

### 5.3. Quadratic Discriminant Algorithm (Q.D.A.)

Those classification algorithms whose decision boundaries are expressed as a quadratic function (circles, ellipses, parabolas, hyperbolas) are known as quadratic classifiers [30]. Generally, the covariance matrices of each class are different, thus the discriminant functions have inherently quadratic decision boundaries and are expressed as a quadratic function of a set of features:*v*. Discriminating functions have the following expression:

(13) $$g_{i}(v)=-\frac{1}{2}{\left(v-\mu_{i}\right)}^{T}\sum_{i}^{-1}\left(v-\mu_{i}\right)-\frac{1}{2}\text{log}\left|\sum_{i}\right|+\text{log}(p(w_{i}))$$

where:

*μ_i_* = *E*(*v*|*w_i_*) is the average vector for the *w_i_* class.
Σ*_i_* is the *w_i_* class covariance matrix.

A set of equivalent discriminating functions for each class can be derived from the previous equation:

(14) $$g_{i}(v)=v^{T}{\bar{k}}_{i}v+k_{i}^{T}v+k_{io}$$

where:

${\bar{k}}_{i}=\frac{1}{2}\sum_{i}^{-1}$
$k_{i}=\sum_{i}^{-1}\mu_{i}$
$k_{io}=-\frac{1}{2}{\left(\mu_{i}\right)}^{T}\sum_{i}^{-1}\mu_{i}-\frac{1}{2}\text{log}|\sum_{i}|+\text{log}(p(w_{i}))$

The same set *v* = {*v*_1_, *v*_2_, *v*_3_, *v*_4_} that in KNN algorithm has been used for classification. A set of 300 training patterns are used for walls, and another 300 for corners. Covariance matrices *k*_1_ and *k*_2_ as well as the *k*_10_ and *k*_20_ constant values were obtained from these datasets. Two discriminating functions are used in order to classify: if *g*1(*v*) ≤ *g*2(*v*) then the obstacle is classified as a corner, otherwise it is classified as a wall.

(15) $$\begin{array}{l} g_{1}(v)=v^{T}{\bar{k}}_{1}v+k_{1}^{T}v+k_{1o}\\ g_{2}(v)=v^{T}{\bar{k}}_{2}v+k_{2}^{T}v+k_{2o} \end{array}$$

## 6. Experimental Results and Discussion

The above classification algorithms described have been applied using the echoes obtained from the YAIR robot (see Figure 1) walking in several rooms and corridors of our school. These measurements have been taken indoor, under different temperature and humidity conditions during about one year in Valencia. As all the scenarios are indoor, the variations in temperature and humidity are reduced (temperature ranges from 19 to 24 degrees and relative humidity ranges from 50% to 70%). Thus, we have not found any appreciable difference between the results obtained for different ambient conditions. For this reason, the results are not presented as a function of them.

The experiments have been organized into two data sets, depending on the material composition of the scenario.

- Data set 1. The scenarios were composed mainly of concrete (*Cr = 0.59*), and others of pladur® (*Cr = 0.62*). Note: The corners were composed exclusively of only one of these two materials at each of the scenarios.
- Data set 2. The scenarios were composed of different kinds of materials in the corners. Walls were mainly made of pladur®, some pillars of cement (*Cr = 0.59*) and pladur®, a door of laminated material (*Cr = 0.64*) with wood doorframes (*Cr = 0.5*), and some metal downspout frames (*Cr = 0.5*), and glass windows (*Cr = 0.71*).

The samples vary in their composition, the proportion of obstacles over short distances (less than 1.5 m) on 300 samples, and the remainder (up to 4 m) between 100 and 200 samples. The incidence angles have also varied from 20 to 70 degrees.

The classification algorithm based on amplitude gives good results at short distances. Table 1 shows results for distances less than 1.5 m. Having best results in uniform scenario composition, using as parameters *Cr* = 0.6 and *N*_0_ = 1.3. No obstacle is classified as unknown—it must be classified always as a wall or a corner. As expected, due to the more challenging nature of the dataset 2, the obtained results for this set are in general worst than those obtained for dataset 1, specially for the corners.

The algorithm of the K nearest neighbors provides different success rates depending on the value of K. The tests show that the higher value of K the higher success rates in corners, but worse in walls. So a compromise must be made in order to obtain the best performance in both types of obstacles. In the Table 2 the accuracy rates for the two datasets are presented for some K values. The optimum results are obtained for K = 10.

The results of algorithm classification based on pattern recognition techniques have been summarised in Table 3. As can be seen, the results are pretty good, getting some average success rates from 80% to 90%. Bearing in mind that obstacles are being located up to distances of 4 m, simply through information provided by ultrasonic echo amplitude, these results are satisfactory. Moreover, improvement is more evident in the detection of corners. The best results are obtained by the KNN algorithm.

## 7. Conclusions

A set of methods for classifying an obstacle previously located have been presented. Classification is performed by taking a single sonar scan, which has been obtained with a single sensor ultrasonic piezo-ceramic type, by applying a model based on signal amplitude and the extraction of geometric features of the environment. The tests presented are taken from many empty rooms. Rooms whose walls are of different materials, mostly concrete (*Cr* = 0.59) and pladur (*Cr* = 0.62). The measurements were taken for distances between 50 cm up to 4 m, for incidence angles ranging from 20° to 70°. An algorithm that consists of applying a model of the ultrasonic amplitude has been presented, and also several methods based on algorithms widely used in pattern recognition, that combine both amplitude and geometric features.

The classification algorithm based on amplitude model offers very good results at short distances, 88% success rate on average in walls and 85% in corners at distances less than 1.5 m. The success rate decreases as distance increases up to 4 m, having 88% in walls and 68% in corners. When adding information from the geometric characteristics, and applying classical algorithms in pattern recognition then the success rate increases. The method that offers better results is the KNN algorithm, for *K* = 10 (see Tables 2 and 3). It provides 90% success rate in walls, and 91% accuracy rate on average in corners, but its algorithmic load is high. It is followed closely by the algorithm based on prototypes of Denoeux, with 95% hit rate on average in walls, and 89% accuracy rate on average in corners, which is very close to previous algorithm, but with a significantly lower algorithmic load, and finally, the quadratic discriminant analysis that obtains an 86% success rate in walls, and 73% accuracy rate on average in corners.

**Figure 8.**
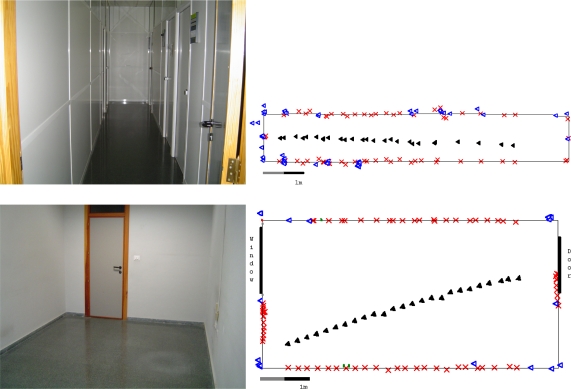
Examples of real environments where experiments were conducted. The resulting maps show the classified walls as red crosses, and the corners as blue triangles. The corresponding positions of the robot are represented as black filled triangles. Top left photo represents a small corridor. Note that the lateral walls have several door frames which are classified as corners in the corresponding map (top right). Down left photo shows an empty room. The corresponding map (down right) obtained shows the results of classification.

The Figure 8 shows some typical scenarios where experiments have been conducted, as well as the resulting maps obtained using geometrical features and KNN algorithm. As can be seen, some errors of classification are produced (mainly in corner misclassification due to long distances) but the overall quality of maps is satisfactory, taken into account that each object has been obtained without any data fusion between successive scans.

## Figures and Tables

**Table 1.** Classification results obtained using amplitude-based algorithm (true positive rates). Distances are lower than 1.5 m.

	Wall	Corner

Dataset 1	88%	85%
Dataset 2	75%	66%

**Table 2.** Classification algorithm KNN results for different values of K. (Distances less than 4m, for all incidence angles). As it can be seen, best results are obtained for *K* = 10.

	K =6		K=10	

	Wall	Corner	Wall	Corner

Dataset 1	92%	88%	90%	91%
Dataset 2	93%	77%	93%	84%

	K=12		K=25	

	Wall	Corner	Wall	Corner

Dataset 1	89%	92%	85%	94%
Dataset 2	92%	84%	90%	87%

**Table 3.** Success percentages obtained for different classification algorithms. For distances under 4 m and all incidence angles.

	Set 1		Set 2	

Algorithm	Wall	Corner	Wall	Corner

Amplitude Based	88%	68%	72%	50%
KNN (*K* = 10)	90%	91%	93%	84%
Denoeux-DS	90%	88%	80%	74%
Q.D.A.	86%	73%	89%	82%
